# Pre-treatment serum apolipoprotein E: a promising prognostic indicator for nasopharyngeal carcinoma

**DOI:** 10.1186/s40001-025-02745-7

**Published:** 2025-06-11

**Authors:** Xian-Ming He, Si-Cong Jiang, Qi-Wei Luo, Jian-Wu Ding, Rong-Huan Hu, Jia-Li Hu, Meng-Meng Liu, Lei Tao, Jian-Ze Zhang, Jia-Yin Wu, Su Deng, Lei Zeng

**Affiliations:** 1 Department of Oncology, The Second Affiliated Hospital, Jiangxi Medical College, Nanchang University, Nanchang, 330006 People’s Republic of China; 2 The Second Affiliated Hospital, Jiangxi Medical College, Nanchang University, Nanchang, 330006 People’s Republic of China; 3 The Rehabilitation College of Nanchang University, Nanchang, Jiangxi Province, 330031, People’s Republic of China

**Keywords:** Nasopharyngeal carcinoma, Apolipoprotein E, Prognosis, Biomarker, Risk stratification

## Abstract

**Background and purpose:**

This preliminary study explores the prognostic value of pretreatment peripheral serum apolipoprotein E (ApoE) in patients with non-metastatic nasopharyngeal carcinoma (NPC).

**Materials and methods:**

A retrospective collection of pretreatment indicators was conducted for 352 nasopharyngeal carcinoma patients between January 2016 and December 2020. Receiver operating characteristic curve analysis, univariate and multivariate Cox proportional hazards analysis were all utilized to assess the correlation between blood ApoE and overall survival (OS), progression-free survival (PFS), distant metastasis-free survival (DMFS) and local recurrence-free survival (LRFS).

**Results:**

A higher baseline serum ApoE level (> 71.5 mg/L) was markedly associated with poorer OS (HR = 2.255, 95% CI 1.232–4.125, *P* = 0.008) and remained an independent prognostic factor in multivariate Cox regression analysis. Additionally, body mass index (BMI), body surface area (BSA), Epstein-Barr virus (EBV)DNA load, and high-density lipoprotein cholesterol (HDL-C) were also identified as independent predictors of nasopharyngeal carcinoma prognosis.

**Conclusion:**

An elevated pretreatment serum ApoE level is indicative of a poorer prognosis for nasopharyngeal carcinoma patients and is independent of other known prognostic factors. These findings highlight the value of ApoE as a potential biomarker for risk stratification and personalized treatment in nasopharyngeal carcinoma.

**Supplementary Information:**

The online version contains supplementary material available at 10.1186/s40001-025-02745-7.

## Background

Nasopharyngeal carcinoma (NPC) is a malignant tumor endemic to Southeast Asia [1]. Comparative to other head and neck tumors, its pathogenesis is notably more complex with distinct regional characteristics. Current research indicates that this is associated with genetic factors such as Epstein-Barr virus (EBV) infection of nasopharyngeal epithelium, and dietary habits [1–3]. With the advancement of radiotherapy technology and multidisciplinary treatments, the local control rate of NPC surpassed 90% [4]. However, due to the inconspicuous location of this disease together with its distinct tendency for lymph node invasion and distant metastasis, more than 70% of patients receive a diagnosis of locoregionally advanced disease at presentation [5]. Furthermore, approximately 20–30% of NPC patients persistently endure local recurrence or distant metastasis despite undergoing standard radiotherapy and chemotherapy [6, 7]. Once this occurs in such patients it often signals a poor prognosis. The causes of death in NPC patients are directly related to local invasion and distant metastasis of the tumor. It is crucial to identify patients at high risk of recurrence and metastasis early on, as compared to the primary lesion, the lesions of local invasion and distant metastasis have significantly reduced sensitivity to radiotherapy and chemotherapy.

Currently, the TNM staging system is predominantly used for risk stratification and prognosis prediction in NPC patients and has unpredictable survival outcomes for NPC patients [8, 9]. Therefore, patients at the same stage often exhibit significant clinical heterogeneity and present different clinical outcomes under the same treatment. To date, plasma EBV DNA level remains the only biomarker with clinical application value for NPC [2, 10]. However, EBV DNA detection is costly, and there are no unified detection standards. There is considerable variability among laboratories, making it difficult to apply to routine detection. Therefore, screening for some inexpensive, objective, and easily detectable markers to supplement the TNM staging system for the prediction of NPC is highly meaningful and beneficial.

In previous studies, blood lipids were principally epidemiologically related to cardiovascular diseases. Today, further research reveals that changes in blood lipid levels are closely associated with the occurrence of cancer and also play a crucial role in the development and progression of tumors [11]. There is a strong connection between alterations in lipid profiles and the development and metastasis of various cancers. Apolipoprotein E (ApoE), encoded by the ApoE gene, is a lipoprotein and the major lipid carrier in the brain [12]. Previous research on ApoE largely focused on the fields of cardiovascular and Alzheimer's disease [13], and it influences a wide range of common cellular processes (e.g., neuroinflammation, degradation, and clearance of amyloid beta, synaptogenesis, membrane repair and remodeling, and neuronal growth) [14, 15]. In recent years however, research on ApoE extends far beyond this, such as, the study by Urquidi et al. exploring the potential of alpha1-antitrypsin and apolipoprotein E (ApoE) in urine as non-invasive biomarkers for detecting bladder cancer [16]. Peng et al. provides a comprehensive analysis of the expression pattern, prognostic significance, and interaction with immune cells of ApoE in gastric cancer [17]. Through integrated bioinformatics analysis and clinical cohort studies, this research found that the expression level of ApoE within gastric cancer tissues is upregulated and is associated with a shorter duration of overall survival. Wu et al. discovered that the expression of ApoE in endometrial cancer tissue is significantly higher than that in normal tissue and is closely related to the histological grade, lymph node metastasis, and FIGO stage of the tumor [18]. Not only that, there are also relevant reports on colorectal cancer, breast cancer, brain tumors, lung adenocarcinoma, and laryngeal cancer [19–23]. In NPC, Xue et al. found that the expression level of apolipoprotein E (ApoE) is significantly increased, demonstrating the high sensitivity and specificity of ApoE in the serum of NPC patients, suggesting that ApoE could potentially be a promising diagnostic biomarker for NPC [24]. This study is the first to retrospectively analyze the prognostic significance of pretreatment serum apolipoprotein E (ApoE) in NPC patients.

## Methods

### Patients

This study retrospectively analyzed 352 nasopharyngeal carcinoma patients that received initial treatment in the Second Affiliated Hospital of Nanchang University between January 2016 and December 2020. A comparative analysis was undertaken to investigate the prognosis outcome between two groups, a higher pretreatment serum ApoE group and a lower pretreatment serum ApoE group. The Ethics Committee at the Second Affiliated Hospital of Nanchang University approved this retrospective research protocol. Inclusion criteria included: (1) Patients with nasopharyngeal malignant tumors initially confirmed by pathological tissue biopsy under nasopharyngoscopy. (2) Patients that completed the entire treatment process. (3) Patients that completed clinical information and follow-up data. Exclusion criteria included: (1) Patients with other tumors or a previous history of anti-tumor treatment prior to treatment. (2) Patients that refused treatment or failed to complete the entire-course intensity-modulated radiotherapy. (3) Pregnant women. (4) Patients with fatty liver, cirrhosis, or severe liver dysfunction. (5) Patients who were lost to follow-up.

The clinical data of participants was collected and organized from the electronic medical record system at the Second Affiliated Hospital of Nanchang University. This data consisted of gender, age at diagnosis, history of smoking, history of hereditary tumors, T classification, N classification, and WHO pathological classification. Treatment information comprised of induction chemotherapy, concurrent chemotherapy along with the doses and regimens of adjuvant chemotherapy. Pretreatment blood biochemical indicators included albumin, lactate dehydrogenase, total cholesterol, triglyceride, high-density lipoprotein cholesterol, low-density lipoprotein cholesterol, apolipoprotein A, apolipoprotein B, and apolipoprotein E. Survival information consisted of survival status, survival time, progression-free survival time, distant metastasis-free survival time and local recurrence-free survival time.

### Treatment regimens

For patients in stages III–IVA, neoadjuvant chemotherapy combined with concurrent chemoradiotherapy with or without adjuvant chemotherapy was administered. The induction chemotherapy and adjuvant chemotherapy regimens comprised the DP regimen (docetaxel 75 mg/m^2^ on the first day, cisplatin/nedaplatin 25 mg/m^2^/day on the first 3 days), the GP regimen (gemcitabine 1000 mg/m^2^/day on days 1 and 8, cisplatin/nedaplatin 25 mg/m^2^/day on the first 3 days), and the TPF regimen (docetaxel 60 mg/m^2^ on the first day, cisplatin 60 mg/m^2^on the first day, fluorouracil 600 mg/m^2^/day as a continous 120h infusion on days 1-5). During concurrent chemoradiotherapy, single-agent cisplatin/nedaplatin chemotherapy was employed, specifically 80 mg/m^2^ once every 3 weeks or 30 mg/m^2^ once a week. Adjuvant chemotherapy was administered following concurrent chemoradiotherapy according to international guidelines. The chemotherapy regimens were repeated every 21 days. Routine blood work and liver and kidney function examinations were conducted regularly. The selection of the chemotherapy regimen and cycle was personalized for each patient based on individual factors such as their physical condition, financial affordability, and treatment intention. Radiotherapy Standards: The treatment of nasopharyngeal carcinoma patients was in accordance with international guidelines. All patients received intensity-modulated radiotherapy (IMRT). The total tumor volume and clinical tumor volume were determined in line with previous guidelines. The PGTVnx was 66-70 Gy, PGTVnd was 64-70 Gy, PTV1 was 60-64 Gy, and PTV2 was 54-56 Gy. In the conventional fractionation mode, irradiation was performed once a day from Monday to Friday, with respite on Saturday and Sunday. A total of 30–33  fractions of target-volume radiotherapy were completed. Active symptomatic treatment was administered for any acute adverse reactions that occurred during radiotherapy in NCP patients, including skin and oral mucosa reactions.

### Follow-up and study endpoints

After completing radiotherapy, all patients were follow-up every three months for the first two years. Between the third and fifth year, follow-up was performed every six months. After 5 years, follow-up was carried out annually. Follow-up methods included outpatient review and telephone consultation. Follow-up appointments conducted included physical examination, nasopharyngoscopy, relevant hematological and imaging examinations. The follow-up deadline was May 15, 2024, and the follow-up time range was 41 to 101 months. The primary study endpoint was overall survival (OS) rate with the secondary study endpoints being progression-free survival (PFS) rate, distant metastasis-free survival rate (DMFS) and local recurrence-free survival rate (LRFS).

### Statistical analysis

Cut-off values for continuous variables were determined using receiver operating characteristic (ROC) curve analysis, with the optimal threshold selected based on the maximal Youden index (sensitivity + specificity−1) to maximize classification performance. For variables including age, BMI, body surface area, albumin (Alb), total cholesterol triglycerides (TCTG), HDL, LDL, apolipoprotein E(ApoE), apolipoprotein A (ApoA), and apolipoprotein B (ApoB), the cut-offs were individually analyzed according to their association with overall survival (OS), ensuring statistical significance (p < 0.05) through rigorous validation. All endpoints were measured from the date of treatment and analyzed using SPSS V17.0 (SPSS Inc., Chicago, IL). The time to the first definitional event was evaluated for the following endpoints: local recurrence-free survival (LRFS), distant failure-free survival (DMF), progression-free survival (PFS), and overall survival (OS). Survival rates were calculated using the Kaplan–Meier method and compared using the log-rank test. A two-tailed p-value less than 0.05 was considered statistically significant. In the univariate Cox proportional hazards regression analysis, variables with p-values < 0.05 were included in the multivariate analysis to control for potential confounding factors. Stepwise regression method (forward selection) was applied in the multivariate analysis, retaining variables with p-values < 0.05 as independent prognostic factors. The Cox proportional hazards model for OS, PFS, LRFS, and DMFS was used to analyze the entire cohort.

## Result

### Clinical outcomes

**Fig. 1:**
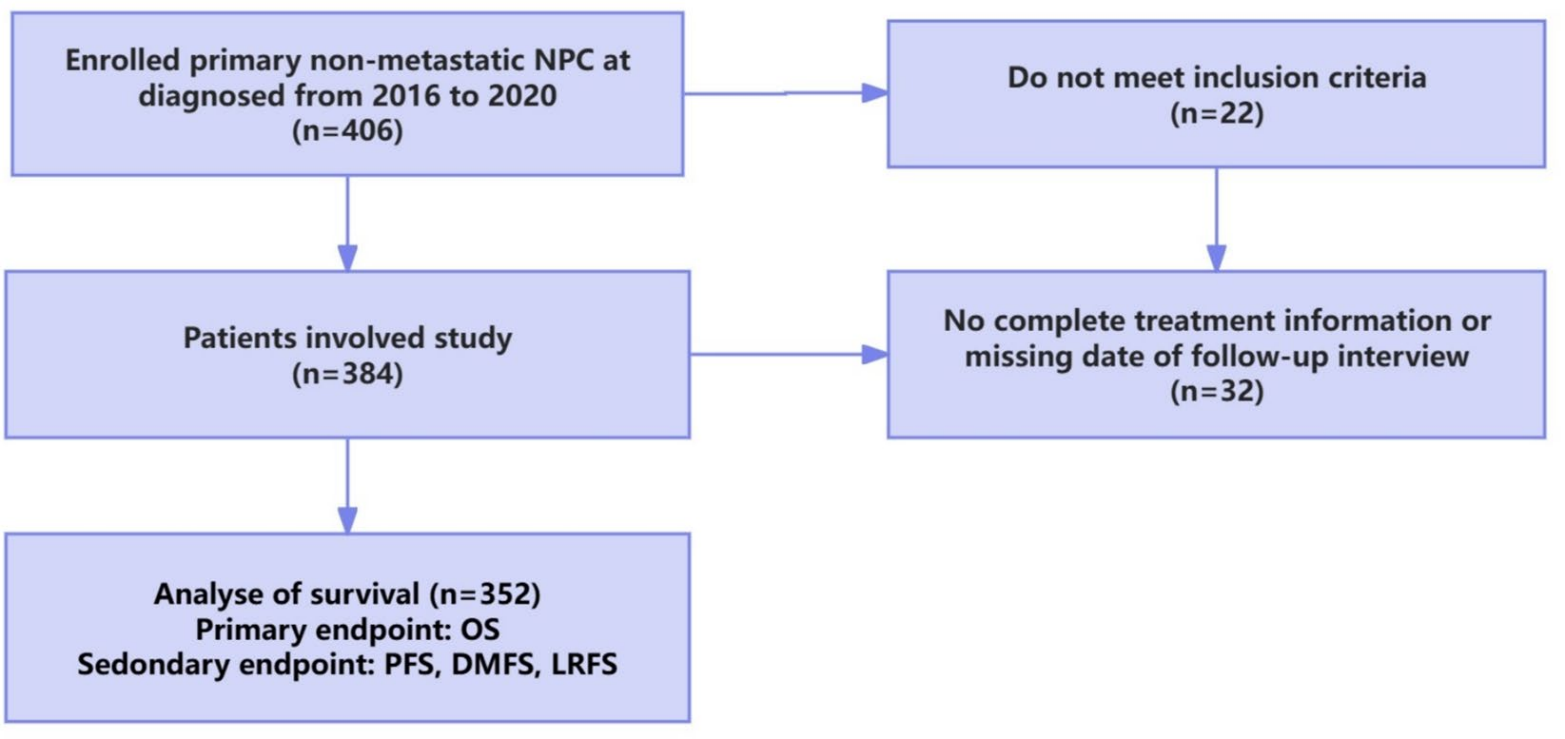
Cohort definition and exclusion. *NPC* nasopharyngeal carcinoma, *PFS* progression-free survival, *OS* overall survival, *DMFS* distant metastasis-free survival, *LRFS*  local recurrence-free survival

A total of 406 cases were enrolled following the inclusion criteria. 54 cases were excluded under the exclusion criteria. Specifically, there were 8 cases with other tumors or a previous history of anti-tumor treatment before diagnosis; 14 cases of patients with fatty liver, cirrhosis or severe liver dysfunction; 10 cases that refused treatment or did not complete radiotherapy; and 22 cases that were lost to follow-up. Ultimately, the total number of cases analyzed in this study was 352. The specific screening process and the research flow of this study are shown in Fig. 1 below. All 352 patients underwent follow-up examinations from their first visit (median, 55 months). During the follow-up period, 65 cases (18.5%) had distant metastasis, 12 cases (3.4%) had local recurrence and 6 cases (1.7%) had both distant metastasis and local recurrence. Almost all of those patients received palliative chemotherapy or radiotherapy. During the follow-up period, 42 (11.9%) patients with recurrent or metastatic disease died. Another 5 patients died without recurrent disease—1 patient died of cerebral infarction, 2 patients died of pulmonary infection, and 2 patients died of complications of radiotherapy—massive bleeding from the nasopharynx. The PFS, OS, DMFS, and LRFS of the 352 patients were 71.9%, 86.4%, 79.2%, and 94.9%, respectively.

### Patient baseline characteristics

As shown in Table 1, for the 352 nasopharyngeal carcinoma patients, with overall survival status as the endpoint and based on the cut-off point determined by the ROC curve, the ApoE level was dichotomized, and the patients were divided into two groups: high ApoE level group (ApoE > 71.5 mg/L) and low ApoE level group (ApoE ≤ 71.5 mg/L). Baseline blood lipid and lipoprotein levels (i.e., triglyceride, cholesterol, HDL-C, LDL-C levels, ApoA-I, and ApoB), body surface area, and BMI were dichotomized according to the cut-off points determined by the ROC curve. Other clinicopathological characteristics (including age, gender, T classification, N classification, and quantitative serum BEV DNA) were subjected to univariate analysis. The chi-square test results indicated that the data is relatively balanced. In the results, the median age of the two patient groups was similar (*P* = 0.187) with the majority being male (74.7% overall,* P* = 0.243). Regarding body mass index (BMI) and body surface area, no significant differences were identified between the two groups (*P* = 0.419 and *P* = 0.329). The T classification distribution showed an insignificant difference (*P* = 0.727), with no significant differences in the selection of treatment regimens (radiotherapy alone or chemoradiotherapy) and the use of adjuvant chemotherapy (p = 0.321, *P* = 0.420). However, in the N classification distribution, there was a substantial difference between the two groups (*P* = 0.019), and a higher proportion of patients in the ApoE > 71.5 mg/L group showed (N2-N3 stage). In addition, a higher proportion of patients in the ApoE > 71.5 mg/L group had an EBV DNA level higher than 1000 copies/mL (*P* = 0.026). In terms of biochemical parameters, triglyceride (TG) levels showed a marked difference between the two groups (*P* = 0.007), and a higher proportion of patients in the ApoE > 71.5 mg/L group had TG levels higher than 3.34 mmol/L. While for total cholesterol (TC), high-density lipoprotein cholesterol (HDL-C), low-density lipoprotein cholesterol (LDL-C), apolipoprotein A-I (ApoA-I), and apolipoprotein B (ApoB) levels, no major differences were observed between the two groups (*P* = 0.256, *P* = 0.314, *P* = 0.869, *P* = 0.555, and *P* = 0.984).

**Table 1:** Demographical characteristics and clinical data of the participating patients

Characteristics	Total patients	Apo-E(mg/L) ≤ 71.5 (n = 273)	Apo-E(mg/L) > 71.5 (n = 79)	*P* value
Age (years)				0.187
≤ 47	108(30.7%)	79(28.9%)	29(36.7%)	
> 47	244(69.3%)	194(71.1%)	50(63.3%)	
Gender				0.243
Male	263(74.7%)	200(73.3%)	63(79.7%)	
Female	89(25.3%)	73(26.7%)	16(20.3%)	
BMI (kg/m^2^)				0.419
≤ 20.8	129(36.6%)	97(35.5%)	32(40.5%)	
> 20.8	223(63.4%)	176(64.5%)	47(59.5%)	
Body surface area (m^2^)				0.329
≤ 1.57	146(41.5%)	117(42.9%)	29(36.7%)	
> 1.57	206(58.5%)	156(57.1%)	50(63.3%)	
EBV DNA (copies/mL)				0.018
≤ 1000	235(67.3%)	191(70.0%)	44(55.7%)	
> 1000	117(32.7%)	82(30.0%)	35(44.3%)	
T classification				0.727
T1–T2	119(33.8%)	91(33.3%)	28(35.4%)	
T3–T4	223(66.2%)	182(66.7%)	51(64.6%)	
N classification				0.019
N0–N1	94(26.7%)	81(29.7%)	13(16.5%)	
N2–N3	258(73.3%)	192(79.3%)	66(83.5%)	
Neoadjuvant chemotherapy				
DP regimen	150(42.6%)	119(43.6%)	31(39.2%)	0.386
GP regimen	128(36.4%)	101(37.0%)	27(34.2%)	
TPF regimen	74(21.0%)	53(19.4%)	21(26.6%)	
Treatment arm				0.321
Radiotherapy alone	161(45.7%)	121(44.3%)	40(50.6%)	
Chemoradiotherapy	191(54.3%)	152(55.7%)	39(49.4%)	
Adjuvant chemotherapy				
Yes	170(48.3%)	135(49.5%)	35(44.3%)	0.420
No	182(51.7%)	138(50.5%)	44(55.3%)	
ALB(g/L)				
≤ 41.6	165(46.9%)	133(48.7%)	32(40.5%)	0.198
> 41.6	187(53.1%)	140(51.3%)	47(59.5%)	
TC (mmol/L)				
≤ 3.55	18(5.10%)	12(4.40%)	6(7.6%)	0.256
> 3.55	334(94.9%)	261(95.6%)	73(92.4%)	
TG (mmol/L)				
≤ 3.34	244(5.1%)	199(72.9%)	45(57.0%)	0.007
> 3.34	108(94.9%)	74(27.1%)	35(43.0%)	
HDL-C (mmol/L)				
≤ 1.01	100(28.4%)	74(27.1%)	26(32.9%)	0.314
> 1.01	252(71.6%)	199(72.9%)	53(67.1%)	
LDL-C (mmol/L)				
≤ 2.3	69(19.6%)	53(19.4%)	16(20.3%)	0.869
> 2.3	283(80.4%)	220(80.6%)	63(79.7%)	
ApoA-I (g/L)				
≤ 1.29	215(61.1%)	169(61.9%)	46(58.2%)	0.555
> 1.29	137(38.9%)	104(38.1%)	33(41.8%)	
ApoB (g/L)				
≤ 0.87	134(38.1%)	104(38.1%)	30(38.0%)	0.984
> 0.87	218(61.9%)	169(61.9%)	49(62.0%)	

### Survival analysis based on ApoE

The 352 patients were divided into two groups according to the pretreatment ApoE level of nasopharyngeal carcinoma patients to analyze their various survival prognoses. The cut-off point of ApoE (71.5 mg/L) was determined by using the optimal diagnostic threshold value obtained from the ROC curve. The results showed that when comprehensively evaluating the 5-year PFS, OS, DMFS, and LRFS, the prognosis of patients with high ApoE levels was worse than that of patients with low ApoE levels. In terms of the 5-year OS rate, the 5-year OS rates of the high and low ApoE groups were 64.5% and 73.1%, respectively (*P* = 0.0016). The 5-year PFS and DMFS were similar to the 5-year OS, having *P* values of *P* = 0.0001 and *P* = 0.0001, respectively. There was no significant difference in the 5-year LRFS.

### Univariate Cox proportional hazards regression analysis of clinicopathologic characteristics

As shown in Table 2, baseline blood lipid and lipoprotein levels (i.e., triglyceride, cholesterol, HDL-C, LDL-C levels, ApoA-I, ApoB, and ApoE), body surface area, and BMI were subjected to univariate analysis according to the cut-off points determined by the ROC curve and other clinicopathological characteristics (including age, gender, T classification, N classification, and serum EBV DNA). Clinical variables related to survival included: body mass index (HR = 0.339, 95% CI 0.188–0.611, *P* < 0.001), body surface area (HR = 0.372, 95% CI 0.205–0.675, *P* = 0.001), EBV DNA level (HR = 2.938, 95% CI 1.645–5.249, *P* = 0.001), N classification (HR = 4.163, 95% CI 1.494–11.60, *P* = 0.006), which are associated with OS, while height is not. Among blood lipid and lipoprotein characteristics, low cholesterol level (HR = 0.386, 95% CI 0.163–0.911, *P* = 0.030), high triglyceride level (HR = 2.286, 95% CI 1.068–4.895, *P* = 0.033), low HDL-C level (HR = 0.243, 95% CI 0.136–0.436, *P* = 0.000), high LDL-C level (HR = 2.008, 95% CI 1.088–3.709, *P* = 0.026), and high ApoE level (HR = 2.481, 95% CI 1.385–4.446, *P* = 0.002) were identified as unfavorable factors for OS. Similarly, 5-year OS combined with 5-year PFS, the following conclusion was identified: high EBV DNA level (HR = 2.029, 95% CI 1.363–3.021, *P* = 0.000), advanced N classification (HR = 1.919, 95% CI 1.138–3.235, *P* = 0.015), high triglyceride level (HR = 1.653, 95% CI 1.029–2.656, *P* = 0.038), and high ApoE level (HR = 2.481, 95% CI 1.385–4.446, *P* = 0.002) may be associated with a poorer prognosis.

**Table 2:** OS Univariate analysis of clinicopathologic characteristics

Characteristics	Univariate analysis			Multivariate analysis		
	HR	95% CI for HR	*P* value	HR	95% CI for HR	*P* value
OS						
Age (years)	1.811	(0.874, 3.752)	0.110			
Gender	0.823	(0.434, 1.561)	0.551			
BMI (kg/m^2^)	0.339	(0.188, 0.611)	0.000	0.426	(0.227, 0.797)	0.008
Body Surface Area (m^2^)	0.372	(0.205, 0.675)	0.001	0.391	(0.204, 0.749)	0.005
EBV DNA (copies/mL)	2.938	(1.645, 5.249)	0.001	2.123	(1.157, 3.894)	0.015
T classification	1.504	(0.223, 1.504)	0.223			
N classification	4.163	(1.494, 11.60)	0.006			
Neoadjuvant chemotherapy	1.030	(0.560, 1.895)	0.923			
Treatment arm	0.585	(0.319, 1.070)	0.082			
Adjuvant chemotherapy	0.965	(0.542, 1.716)	0.903			
TC (mmol/L)	0.386	(0.163, 0.911)	0.030			
TG (mmol/L)	2.286	(1.068, 4.895)	0.033			
HDL-C (mmol/L)	0.243	(0.136, 0.436)	0.000	0.198	(0.107, 0.367)	0.000
LDL-C (mmol/L)	2.008	(1.088, 3.709)	0.026			
ApoA-I (g/L)	0.617	(0.348, 1.095)	0.099			
ApoB (g/L)	1.505	(0.849, 2.668)	0.162			
ApoE (mg/L)	2.481	(1.385, 4.446)	0.002	2.255	(1.232, 4.125)	0.008
PFS						
Age (years)	1.334	(0.847, 2.099)	0.213			
Gender	0.947	(0.594, 1.510)	0.819			
BMI (kg/m^2^)	0.502	(0.388, 0.744)	0.001	0.565	(0.370, 0.864)	0.008
Body Surface Area (m^2^)	0.733	(0.494, 1.088)	0.124			
EBV DNA (copies/mL)	2.029	(1.363, 3.021)	0.000	1.690	(1.114, 2.562)	0.014
T classification	1.019	(0.671, 1.547)	0.930			
N classification	1.919	(1.138, 3.235)	0.015	1.770	(1.042, 3.004)	0.035
Neoadjuvant chemotherapy	0.999	(0.636, 1.568)	0.995			
Treatment arm	0.716	(0.478, 1.072)	0.105			
Adjuvant chemotherapy	0.936	(0.630, 1.388)	0.741			
TC (mmol/L)	0.436	(0.227, 0.838)	0.013			
TG (mmol/L)	1.653	(1.029, 2.656)	0.038	1.756	(1.059, 2.911)	0.029
HDL-C (mmol/L)	0.491	(0.329, 0.733)	0.000	0.453	(0.298, 0.689)	0.000
LDL-C (mmol/L)	1.115	(0.688, 1.805)	0.659			
ApoA-I (g/L)	0.855	(0.573, 1.276)	0.440			
ApoB (g/L)	1.225	(0.823, 1.824)	0.316			
ApoE (mg/L)	2.881	(1.922, 4.321)	0.000	2.556	(1.667, 3.918)	0.000

Table 2. NPC, nasopharyngeal carcinoma; HR, hazard ratio; CI, confidence interval; PFS, progression-free survival; OS, overall survival; BMI, body mass index; EBV, Epstein-Barr virus; TC, total cholesterol; TG, triglycerides; HDL-C, high-density lipoprotein cholesterol; LDL-C, low-density lipoprotein cholesterol; ApoA-I, apolipoprotein A-I; ApoB, apolipoprotein B; ApoE, apolipoprotein E. Hazard ratios and p-values with an adjusted multivariate Cox proportional hazards regression model, including age (> 47 years vs. ≤ 46 years), sex (male vs. female), BMI (> 20.8 kg/m^2^ vs. ≤ 20.8 kg/m^2^), Body Surface Area (> 1.57 m^2^ vs. ≤ 1.57 m^2^), EBV DNA (> 1000 copies/mL vs. ≤ 1000 copies/mL), T classification (T1 + T2 vs. T3 + T4), N classification (N0 + N1 vs. N2 + N3), Neoadjuvant Chemotherapy (DP regimen vs. GP regimen vs. TPF regimen), Treatment Arm (Radiotherapy alone vs. Chemoradiotherapy), Adjuvant Chemotherapy (yes vs. no), TC level (> 3.55 mmol/L vs. ≤ 3.55 mmol/L), TG level (> 3.34 mmol/L vs. ≤ 3.34 mmol/L), HDL-C level (> 1.01 mmol/L vs. ≤ 1.01 mmol/L), LDL-C level (> 2.3 mmol/L vs. ≤ 2.3 mmol/L), ApoA-I level (> 1.29 g/L vs. ≤ 1.29 g/L), ApoB level (> 0.87 g/L vs. ≤ 0.87 g/L), ApoE level (> 71.5 mg/L vs. ≤ 71.5 mg/L) were calculated as covariates. Variables were selected with the forward stepwise approach. Only variables that were significantly associated with survival are presented.

### Results of multivariate analysis

**Fig. 2:**
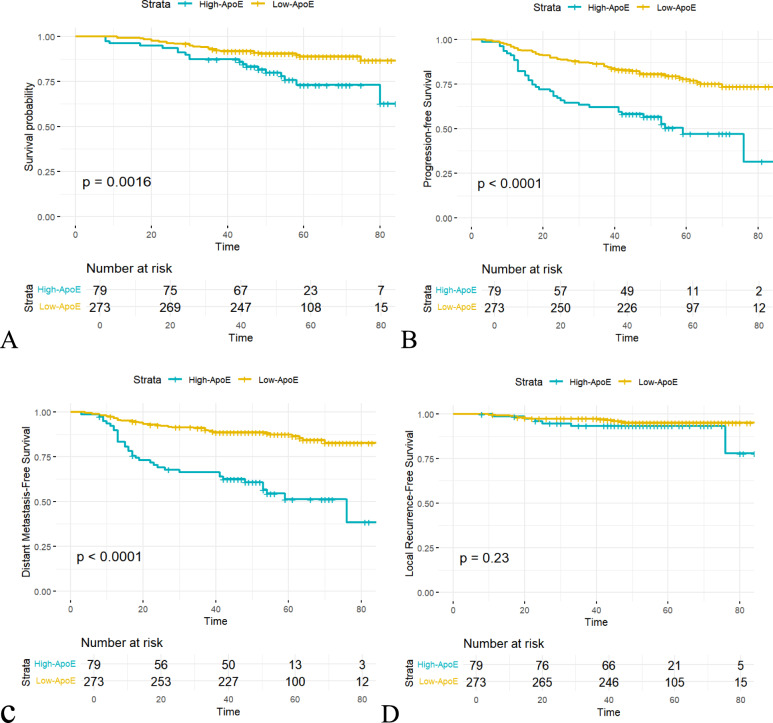
Kaplan–Meier curves of progression-free survival according to different groups in 352 NPC patients. **A** Overall survival; **B** Progression-free Survival; **C** Distant metastasis-free survival; **D** Local recurrence-free survival

In an endeavor to further scrutinize the influence of diverse clinicopathological characteristics on the prognosis of nasopharyngeal carcinoma patients, a multivariate survival analysis was undertaken to control for confounding factors and appraise the independent relationship between each variable and prognosis. The findings revealed that an elevated ApoE level served as a significant independent predictor of adverse OS (> 71.5 mg/L versus ≤ 71.5 mg/L), with HR = 2.255, 95% CI 1.232–4.125, *P* = 0.008 (Table 2). ApoE remained a prognostically significant factor both clinically and statistically (Fig. 2). Additionally, body mass index (HR = 0.426, 95% CI 0.227–0.797, *P* < 0.001), body surface area (HR = 0.391, 95% CI 0.204–0.749, *P* = 0.005), EBV DNA (HR = 2.123, 95% CI 1.157–3.894, *P* = 0.015), and HDL-C (HR = 0.198, 95% CI 0.107–0.367, *P* = 0.000) were also recognized as independent predictors that impact the prognosis of nasopharyngeal carcinoma patients.

## Discussion

This study represents the first large-scale cohort exploration of the relationship between ApoE within blood lipids and the prognosis of nasopharyngeal carcinoma. In this research, an elevated baseline ApoE level was found to be highly associated with poorer OS and was demonstrated to be independent of other variables that serve as predictors for the prognosis of patients with metastatic nasopharyngeal carcinoma. Additionally, pretreatment BMI, body surface area, EBV DNA, and HDL-C were identified as independent prognostic indicators for disseminated nasopharyngeal carcinoma.

This study is in congruence with the previously established correlations between EBV DNA, BMI, and the prognosis of nasopharyngeal carcinoma. Moreover, akin to the study by Yao et al. in 2018, it was discovered that the baseline high-density lipoprotein (HDL) level constitutes an independent favorable prognostic factor for overall survival and progression-free survival in patients with NPC[25]. Apolipoprotein E (ApoE), a protein with multifarious functions within the human body [26], plays a particularly crucial role in the transportation and metabolism of lipids [27]. Expressed in multiple cell types including those of the liver, brain, and immune system, ApoE has three main allelic variants—ε2, ε3, and ε4—which are determined by single nucleotide polymorphisms (SNPs) in the gene [19, 28]. These distinct variants exhibit slight differences in amino acid sequence, thereby resulting in variations in their structure and function. Additionally, ApoE serves as a major brain cholesterol carrier and is the strongest risk locus for late-onset Alzheimer's disease, facilitating the deposition and accumulation of amyloid plaques. Previous research on ApoE predominantly focused on Alzheimer's disease and the cardiovascular field [29–31]. However, as investigations into the mechanisms related to malignant tumors in lipid metabolism have progressively increased over the years, attention has been directed towards the relationship between ApoE and cancer. Recent in-depth studies of ApoE revealed that the expression level of ApoE is upregulated in most cancers and is significantly correlated with the prognosis of patients. For instance, Hui B, Lu C, Li H, et al. (2022) explored the dual role of ApoE in cancer immunotherapy and found that the inhibition of ApoE can enhance the efficacy of immune checkpoint therapy [23]. ApoE overexpression, mediated through interaction with the LRP1 receptor, can promote the migration and invasion of CRC cells [22]. Liu et al. via serum-free cell culture and proteomic analysis, uncovered the key differentially expressed protein ApoE in the secretome. Its expression in lung adenocarcinoma cells with lymph node metastasis is significantly higher than that in cells without metastasis, further suggesting its potential as an indicator of lung adenocarcinoma metastasis [32]. Nevertheless, studies on the relationship between ApoE and the prognosis of nasopharyngeal carcinoma remain unreported. This study indicates that a higher baseline ApoE level is not only closely linked to poorer OS but also constitutes an independent prognostic factor in multivariate analysis under the Cox model. However, the mechanism underlying the relationship between ApoE level and cancer development remains elusive. Studies also show that ApoE is a highly specific and effective protein in exosomes derived from M2-type TAMs. The exosomes of TAMs can enhance the migration ability of gastric cancer cells by activating the PI3 K-Akt signaling pathway [17]. Liu et al.'s research on ApoE and immunity-related studies suggested that the interaction between ApoE + macrophages and CD8 + exhausted T cells is a key factor affecting the immune treatment response. This interaction may lead to Tex cells being unable to effectively conduct immune surveillance and clearance of tumor cells. By secreting specific cytokines and chemokines such as CCL2, ApoE, and TIMP1, it promotes the invasion and metastasis of tumor cells [20]. Additionally, Wan et al. recently found that ApoE plays an important role in the metastasis and sorafenib resistance of hepatocellular carcinoma by promoting cholesterol accumulation, accelerating the formation of lipid rafts, and activating the PI3 K-AKT signaling pathway. Further research is imperative to elucidate these mechanisms.

This study has certain limitations. Firstly, it is a retrospective analysis, so there may be potential sources of bias. Subsequently, it is a single-center study on a limited number of patients and the data is sourced from one research center. Nasopharyngeal carcinoma is a malignant tumor with obvious regional characteristics and is characterized by an unbalanced local distribution. The research center in this study is located within an area of high incidence of nasopharyngeal carcinoma. It cannot be determined whether the results of this study can be extrapolated to areas with a lower incidence of nasopharyngeal carcinoma. Therefore, these research results should be interpreted with caution until they are verified in a large multi-institutional pooled analysis. Furthermore, the ApoE cut-off value (71.5 mg/L) identified in this study was determined based on ROC curve analysis from our cohort using our institutional assay methodology. Variations in detection techniques across different laboratories (e.g., reagents, calibration standards, or analytical platforms) may influence the reproducibility of this threshold. To enhance the clinical utility of serum ApoE as a prognostic biomarker, future investigations should focus on establishing standardized assay protocols that include uniform reagents and calibration methodologies, thereby ensuring comparability across diverse clinical settings.

Finally, there is currently no unified standard for the selection of cut-off values for blood lipid levels. In this study, the selection of cut-off values was based on the judgment of the maximum Youden index in the data, which may contain certain errors. Moreover, other prognostic indicators such as inflammatory markers and radiomics data are not included.

In conclusion, it is believed that this study is the first retrospective undertaking to investigate the relationship between baseline ApoE and nasopharyngeal carcinoma. The research results indicate that an elevated pretreatment serum APOE level is an independent prognostic factor for poorer overall survival in our cohort. In addition, it demonstrated that baseline BMI and body surface area affect the survival of nasopharyngeal carcinoma patients. Therefore, further multicenter prospective studies are needed. The specific mechanism by which ApoE affects the prognosis of nasopharyngeal carcinoma remains unclear and further exploration is required in future studies.

## Supplementary Information

Additional file1

## Data Availability

No datasets were generated or analysed during the current study.

## References

[1] Chen Y-P, Chan ATC, Le Q-T, Blanchard P, Sun Y, Ma J. Nasopharyngeal carcinoma. The Lancet. 2019;394(10192):64–80. 10.1016/S0140-6736(19)30956-0 31178151

[2] Young LS, Dawson CW. Epstein-Barr virus and nasopharyngeal carcinoma. Chin J Cancer. 2014;33:581–90. 25418193 10.5732/cjc.014.10197 PMC4308653

[3] Zheng S, Matskova L, Zhou X, Xiao X, Huang G, Zhang Z, Ernberg I. Downregulation of adipose triglyceride lipase by EB viral-encoded LMP2A links lipid accumulation to increased migration in nasopharyngeal carcinoma. Mol Oncol. 2020;14(12):3234–52. 33064888 10.1002/1878-0261.12824 PMC7718958

[4] Chen L, Hu C-S, Chen X-Z, Hu G-Q, Cheng Z-B, Sun Y, Li W-X, Chen Y-Y, Xie F-Y, Liang S-B, et al. Concurrent chemoradiotherapy plus adjuvant chemotherapy versus concurrent chemoradiotherapy alone in patients with locoregionally advanced nasopharyngeal carcinoma: a phase 3 multicentre randomised controlled trial. Lancet Oncol. 2012;13(2):163–71. 22154591 10.1016/S1470-2045(11)70320-5

[5] Mao Y-P, Xie F-Y, Liu L-Z, Sun Y, Li L, Tang L-L, Liao X-B, Xu H-Y, Chen L, Lai S-Z, Lin A-H, Liu M-Z, Ma J. Re-evaluation of 6th edition of AJCC staging system for nasopharyngeal carcinoma and proposed improvement based on magnetic resonance imaging. Int J Radiat Oncol Biol Phys. 2009; 73(5):1326–34. 10.1016/j.ijrobp.2008.07.062 19153016

[6] Chen Y-P, Ismaila N, Chua MLK, Colevas AD, Haddad R, Huang SH, Wee JTS, Whitley AC, Yi J-L, Yom SS, et al. Chemotherapy in combination with radiotherapy for definitive-intent treatment of stage II–IVA nasopharyngeal carcinoma: CSCO and ASCO Guideline. J Clin Oncol. 2021;39(7):840–59. 33405943 10.1200/JCO.20.03237

[7] Le QT, Colevas AD, O’Sullivan B, Lee AWM, Lee N, Ma B, Siu LL, Waldron J, Lim C-M, Riaz N, et al. Current treatment landscape of nasopharyngeal carcinoma and potential trials evaluating the value of immunotherapy. JNCI J Natl Cancer Inst. 2019;111(7):655–63. 30912808 10.1093/jnci/djz044 PMC7962891

[8] Zhang W-R, Du Y-Y, Guo C-Y, Zhou H-X, Lin J-Y, Meng X-H, Mo H-Y, Luo D-H. Prognostic value of serum Epstein-Barr virus antibodies and their correlation with TNM classification in patients with locoregionally advanced nasopharyngeal carcinoma. Cancer Res Treat. 2021;53(4):991–1003. 33494127 10.4143/crt.2020.1298 PMC8524010

[9] Hui EP, Li WF, Ma BB, Lam WKJ, Chan KCA, Mo F, Ai QYH, King AD, Wong CH, Guo R, et al. Integrating postradiotherapy plasma Epstein-Barr virus DNA and TNM stage for risk stratification of nasopharyngeal carcinoma to adjuvant therapy. Ann Oncol. 2020;31(6):769–79. 32217076 10.1016/j.annonc.2020.03.289

[10] Mizokami H, Okabe A, Choudhary R, Mima M, Saeda K, Fukuyo M, Rahmutulla B, Seki M, Goh B-C, Kondo S, et al. Enhancer infestation drives tumorigenic activation of inactive B compartment in Epstein-Barr virus-positive nasopharyngeal carcinoma. EBioMedicine. 2024;102:105057. 38490101 10.1016/j.ebiom.2024.105057 PMC10951899

[11] Sung H, Siegel RL, Torre LA, Pearson-Stuttard J, Islami F, Fedewa SA, Goding Sauer A, Shuval K, Gapstur SM, Jacobs EJ, et al. Global patterns in excess body weight and the associated cancer burden. CA A Cancer J Clin. 2018;69(2):88–112. 10.3322/caac.21499 30548482

[12] Shi Y, Andhey PS, Ising C, Wang K, Snipes LL, Boyer K, Lawson S, Yamada K, Qin W, Manis M, et al. Overexpressing low-density lipoprotein receptor reduces tau-associated neurodegeneration in relation to apoE-linked mechanisms. Neuron. 2021;109(15):2413-2426.e2417. 34157306 10.1016/j.neuron.2021.05.034 PMC8349883

[13] Zhao N, Liu C-C, Qiao W, Bu G. Apolipoprotein E, receptors, and modulation of Alzheimer’s Disease. Biol Psychiat. 2018;83(4):347–57. 28434655 10.1016/j.biopsych.2017.03.003 PMC5599322

[14] Yu S, Qian L, Ma JUN. Comprehensive analysis of the expression and prognosis for APOE in malignancies: a pan-cancer analysis. Oncol Res. 2022;30(1):13–22. 37304007 10.32604/or.2022.026141 PMC10207989

[15] Bu G. APOE targeting strategy in Alzheimer’s disease: lessons learned from protective variants. Mol Neurodegenerat. 2022;17(1):51. 10.1186/s13024-022-00556-6 PMC9351235 35922805

[16] Urquidi V, Goodison S, Ross S, Chang M, Dai Y, Rosser CJ. Diagnostic potential of urinary α1-antitrypsin and apolipoprotein E in the detection of bladder cancer. J Urol. 2012;188(6):2377–83. 23088986 10.1016/j.juro.2012.07.094 PMC4013779

[17] Peng X, Cai Z, Chen D, Ye F, Hong L. Prognostic significance and immune characteristics of APOE in gastric cancer. Aging. 2023;15(23):13840–53. 38054821 10.18632/aging.205265 PMC10756126

[18] Wu C, Li T, Cheng W. The correlation between APOE expression and the clinical characteristics and prognosis of patients with endometrial cancer. Medicine. 2022;101(37):30536. 10.1097/MD.0000000000030536 PMC9478276 36123916

[19] Butterbrod E, Sitskoorn M, Bakker M, Jakobs B, Fleischeuer R, Roijers J, Rutten GJ, Gehring K. The APOE ε4 allele in relation to pre- and postsurgical cognitive functioning of patients with primary brain tumors. Eur J Neurol. 2021;28(5):1665–76. 33342004 10.1111/ene.14693 PMC8247965

[20] Liu C, Xie J, Lin B, Tian W, Wu Y, Xin S, Hong L, Li X, Liu L, Jin Y, et al. Pan-cancer single-cell and spatial-resolved profiling reveals the immunosuppressive role of APOE+ macrophages in immune checkpoint inhibitor therapy. Adv Sci. 2024;11(23):2401061. 10.1002/advs.202401061 PMC11186051 38569519

[21] Kemp SB, Carpenter ES, Steele NG, Donahue KL, Nwosu ZC, Pacheco A, Velez-Delgado A, Menjivar RE, Lima F, The S, et al. Apolipoprotein E promotes immune suppression in pancreatic cancer through NF-κB–mediated production of CXCL1. Can Res. 2021;81(16):4305–18. 10.1158/0008-5472.CAN-20-3929 PMC8445065 34049975

[22] He L, Shi M, Ren S, Zhang J, Tian Y, Yang X, Liu H. Jun-APOE-LRP1 axis promotes tumor metastasis in colorectal cancer. Biomol Biomed. 2023;23:1026. 37310025 10.17305/bb.2023.9248 PMC10655886

[23] Hui B, Lu C, Li H, Hao X, Liu H, Zhuo D, Wang Q, Li Z, Liu L, Wang X, et al. Inhibition of APOE potentiates immune checkpoint therapy for cancer. Int J Biol Sci. 2022;18(14):5230–40. 36147474 10.7150/ijbs.70117 PMC9461658

[24] Xue Y, Huang S, Huang J, Li S, Zhang C, Zhou X. Identification of apolipoprotein E as a potential diagnostic biomarker of nasopharyngeal carcinoma. Cancer Manage Res. 2020;12:8943–50. 10.2147/CMAR.S239479 PMC7522425 33061590

[25] Yao J-J, He X-J, Lawrence Wayne R, Zhang W-J, Kou J, Zhang F, Zhou G-Q, Wang S-Y, Sun Y. Prognostic value of circulating lipoprotein in patients with locoregionally advanced nasopharyngeal carcinoma. Cell Physiol Biochem. 2018;48(1):285–92. 30011397 10.1159/000491728

[26] Ying Z, Tramper N, Zhou E, Boon MR, Rensen PCN, Kooijman S. Role of thermogenic adipose tissue in lipid metabolism and atherosclerotic cardiovascular disease: lessons from studies in mice and humans. Cardiovasc Res. 2023;119(4):905–18. 35944189 10.1093/cvr/cvac131 PMC10153643

[27] Kothapalli D, Liu S-L, Bae Yong H, Monslow J, Xu T, Hawthorne Elizabeth A, Byfield Fitzroy J, Castagnino P, Rao S, Rader Daniel J, et al. Cardiovascular protection by ApoE and ApoE-HDL linked to suppression of ECM gene expression and arterial stiffening. Cell Rep. 2012;2(5):1259–71. 23103162 10.1016/j.celrep.2012.09.018 PMC3535179

[28] Serrano-Pozo A, Das S, Hyman BT. APOE and Alzheimer’s disease: advances in genetics, pathophysiology, and therapeutic approaches. Lancet Neurol. 2021;20(1):68–80. 33340485 10.1016/S1474-4422(20)30412-9 PMC8096522

[29] Stampfer MJ. Cardiovascular disease and Alzheimer’s disease: common links. J Intern Med. 2006;260(3):211–23. 16918818 10.1111/j.1365-2796.2006.01687.x

[30] Lanfranco MF, Ng CA, Rebeck GW. ApoE lipidation as a therapeutic target in Alzheimer’s Disease. Int J Mol Sci. 2020;21(17):6336. 32882843 10.3390/ijms21176336 PMC7503657

[31] Wang C-T, Chen M-Y, Guo X, Guo L, Mo H-Y, Qian C-N, Wen B-X, Hong M-H, Huang P-Y. Association between pretreatment serum high-density lipoprotein cholesterol and treatment outcomes in patients with locoregionally advanced nasopharyngeal carcinoma treated with chemoradiotherapy: findings from a randomised trial. J Cancer. 2019;10(16):3618–23. 31333778 10.7150/jca.32621 PMC6636306

[32] Liu Z, Gao Y, Hao F, Lou X, Zhang X, Li Y, Wu D, Xiao T, Yang L, Li Q, et al. Secretomes are a potential source of molecular targets for cancer therapies and indicate that APOE is a candidate biomarker for lung adenocarcinoma metastasis. Mol Biol Rep. 2014;41(11):7507–23. 25098600 10.1007/s11033-014-3641-4

