# Correction: Pre-treatment serum apolipoprotein E: a promising prognostic indicator for nasopharyngeal carcinoma

**DOI:** 10.1186/s40001-025-03076-3

**Published:** 2025-08-23

**Authors:** Xian-Ming He, Si-Cong Jiang, Qi-Wei Luo, Jian-Wu Ding, Rong-Huan Hu, Jia-Li Hu, Meng-Meng Liu, Lei Tao, Jian-Ze Zhang, Jia-Yin Wu, Su Deng, Lei Zeng

**Affiliations:** 1 Department of Oncology, The Second Affiliated Hospital, Jiangxi Medical College, Nanchang University, Nanchang, 330006 People’s Republic of China; 2 The Second Affiliated Hospital, Jiangxi Medical College, Nanchang University, Nanchang, 330006 People’s Republic of China; 3 The Rehabilitation College of Nanchang University, Nanchang, 330031 Jiangxi People’s Republic of China

**Correction: European Journal of Medical Research (2025) 30:473** 10.1186/s40001-025-02745-7

In the sentence beginning ‘All endpoints were measured from the date of…. ' in this article, the text ‘randomization’ should have read "treatment '.

The original article has been corrected.

