# Supplementary material for: Pre-treatment serum apolipoprotein E: a promising prognostic indicator for nasopharyngeal carcinoma
Source: Eur J Med Res. 2025 Jun 11;30:473. doi: 10.1186/s40001-025-02745-7 (PMC12153166; doi:10.1186/s40001-025-02745-7)
Supplement: Supplementary file 1 — Additional file1 [file 40001_2025_2745_MOESM1_ESM.pdf]

CERTIFICATE OF ENGLISH LANGUAGE EDITING

MANUSCRIPT TITLE:

Serum Apolipoprotein E Pre-Treatment - A Promising Prognostic Indicator For Nasopharyngeal Carcinoma

AUTHORS:

Xian-Ming He, Si-Cong Jiang, Qi-Wei Luo, Jian-Wu Ding, Rong-Huan Hu, Jia-Li Hu, Meng-Meng Liu, Lei Tao, Jian-Ze Zhang, Lei Zeng

16th November 2024

DATE OF SUBMISSION

KERRY HOWELL-SMITH, ELP

khsproofeditor@outlook.com

This document certifies that the manuscript listed above has been edited by a native English Language Professional (ELP) concerning grammar, punctuation, syntax, spelling, and clarity. The research content and the author/s intention have not been altered during the editing process and editorial control over the submitted document is at their discretion. Journal editors can contact the ELP for the edited copy that was sent directly to the author/s using the code ApoECHN1
